# 3D Printing of Cell Culture Devices: Assessment and Prevention of the Cytotoxicity of Photopolymers for Stereolithography

**DOI:** 10.3390/ma13133011

**Published:** 2020-07-06

**Authors:** Sebastian Kreß, Roland Schaller-Ammann, Jürgen Feiel, Joachim Priedl, Cornelia Kasper, Dominik Egger

**Affiliations:** 1 Institute of Cell and Tissue Culture Technologies, Department of Biotechnology, University of Natural Resources and Life Sciences, Vienna, Muthgasse 18, 1190 Vienna, Austria; sebastian.kress@boku.ac.at (S.K.); cornelia.kasper@boku.ac.at (C.K.); 2 Health—Institute for Biomedicine and Health Sciences, JOANNEUM RESEARCH Forschungsgesellschaft mbH, Neue Stiftingtalstrasse 2, 8010 Graz, Austria; roland.schaller-ammann@joanneum.at (R.S.-A.); juergen.feiel@joanneum.at (J.F.); Joachim.Priedl@joanneum.at (J.P.)

**Keywords:** biocompatibility, rapid prototyping, photopolymers, cell culture devices, 3D printing, stereolithography, mesenchymal stem cells

## Abstract

3D printing is increasingly important for the rapid prototyping of advanced and tailor-made cell culture devices. In this context, stereolithography represents a method for the rapid generation of prototypes from photocurable polymers. However, the biocompatibility of commercially available photopolymers is largely unknown. Therefore, we evaluated the cytotoxicity of six polymers, two of them certified as biocompatible according to ISO 10993-5:2009, and we evaluated, if coating with Parylene, an inert polymer widely used in medical applications, might shield cells from the cytotoxic effects of a toxic polymer. In addition, we evaluated the processability, reliability, and consistency of the details printed. Human mesenchymal stem cells (MSCs) were used for cytotoxicity testing as they are widely used and promising for numerous applications in regenerative medicine. MSCs were incubated together with printed photopolymers, and the cytotoxicity was assessed. All photopolymers significantly reduced the viability of MSCs while the officially biocompatible resins displayed minor toxic effects. Further, coating with Parylene completely protected MSCs from toxic effects. In conclusion, none of the tested polymers can be fully recommended for rapid prototyping of cell culture devices. However, coating with Parylene can shield cells from toxic effects and thus might represent a viable option until more compatible materials are available.

## 1. Introduction

Rapid prototyping (RP) is becoming increasingly important for the development of advanced or tailor-made cell culture devices [1]. With materials such as stainless steel or polyether ether ketone (PEEK), the production is relatively slow and expensive, because parts are usually designed as lathe or computer numerical control (CNC) milled parts. Most cytocompatible plastics can be designed as injection-molded parts, but this causes high costs for the injection molds needed per iteration step and means a considerable time delay. Both manufacturing pathways can only be obtained from third-party suppliers. In contrast, RP by 3D printing from computer-aided design (CAD) models has enabled researchers to generate parts or devices in a short period of time. Moreover, more and more open source projects enable an affordable and easy access to 3D printing [2]. In the context of cell culture devices, 3D printing is widely used to construct microfluidic devices [3,4] or scaffolds for tissue engineering [5,6], but has also been used to print customized cell culture lab ware [7]. However, 3D printing is becoming also increasingly important for the RP of advanced or tailor-made cell culture devices [8]. For example, miniaturized bioreactor systems for cell expansion or tissue engineering applications are valuable to save resources and increase reproducibility [9]. Furthermore, cell culture devices, such as bioreactors or screening platforms, tailored for the generation of relevant 3D tissue models for in vitro testing are of increasing interest [10]. In this context, additive manufacturing by stereolithography (SLA) is an affordable method that enables the rapid generation of prototypes. Optimally, photopolymers for this process should withstand high temperatures to sustain steam-sterilization at 121 °C for 20 min at 2 bar (autoclavation) [11], enable printing of complex structures such as sealings or mountings (high resolution, <50 µm layer thickness), be transparent to enable the performance of optical microscopy, and be biocompatible (no cytotoxic effects) [12]. For example, parts for tailor-made cell culture devices or bioreactors for tissue engineering are often exposed to culture medium for several days or weeks [13]. Thus, any leachables get in direct contact with the cultured cells. Furthermore, construction parts have other requirements than materials for scaffolding in tissue engineering where the induction of biological effects is often wanted [14]. In contrast to that, construction parts should be inert and prevent cell adhesion as this would cause uncontrolled growth and interfere with the culture process [15]. Currently, a photopolymer that fulfils the abovementioned criteria is not available.

Only a very limited number of commercially available photo-polymerizing resins are currently certified as biocompatible (i.e., according to ISO 10993-5:2009 or USP class testing). As manufacturers only declare compounds of known hazardous risks in the material safety data sheet, the complete composition of the resins is usually not available. However, it has been estimated that typical resins contain more than 20 compounds and many known photoinitiators, which are necessary for SLA, are cytotoxic and have been found to remain in the printed parts [16]. In a comprehensive screening of a set of resins for fused deposition modelling (FDM), multi-jet modelling (MJM), and SLA, none of the resins compatible with SLA were biocompatible [17]. A resin that has been certified as biocompatible according to USP Class VI was even identified as toxic in a multispecies toxicity test. Interestingly this effect was observed later than the standard exposure time of 24 h (according to ISO 10993-5) [17]. Thus, the biocompatibility of commercial resins must be assessed for each application separately, even if a resin has already been certified biocompatible [18].

Further, as the dimensions of the device decrease, the ratio of the surface (device) to volume (culture medium) increases, and cells that are cultured in these small devices are exposed to higher amounts of the printed polymer. Adverse cytotoxic effects in systems with a high surface-to-volume ratio might occur, although a material was certified biocompatible. Therefore, the biocompatibility of resins for 3D printing becomes increasingly significant for small or miniaturized systems.

However, one option to prevent the appearance of cytotoxic effects of a polymer is to coat the printed parts with a biocompatible material. For example, coating with Parylene has been reported to reduce the toxicity of a resin on Madin–Darby canine kidney (MDCK) and cultured human keratinocyte (HaCaT) cell lines [19]. Parylene describes a family of polymers that can be vapor-deposited as a coating on a variety of materials. It represents very good barrier qualities as very thin (500 Angstrom), pin-hole-free, still highly conformal films can be deposited. Parylene is biocompatible, inert, hydrophobic, and chemically resistant, which make it an excellent candidate for coatings of biomedical applications [20,21,22]. Furthermore, it is applicable on a wide variety of substrate materials and appears as a non-porous coating when the layer is thick enough (approximately > 0.6 µm). By performing the coating with a polymer gas, even very small details are reliably coated.

For this study we tested six photopolymers that might represent a suitable compromise between the required material properties of photopolymers for stereolithography-based rapid prototyping of cell culture devices. We studied the printing accuracy of the resins, as it is very important for small complex structures, and further, we assessed if the resins have cytotoxic effects by using two different viability assays and viability stainings. Finally, we tested if a coating with Parylene after the printing process is feasible and if a Parylene coating can protect cells from the cytotoxic effects of a toxic polymer.

Previous cytotoxicity testing of photopolymers has mostly been performed with immortalized cell lines or in non-human species, which are not relevant for applications of printed cell culture devices in regenerative medicine [17,19,23,24,25]. Cell lines are commercially available and ensure a high reproducibility of large numbers of assays performed. However, cell lines have limitations considering their application for functional tissue-specific test systems compared to primary cells [26,27,28,29]. Therefore, we used primary human mesenchymal stem cells (MSCs), which are multipotent cells with a high proliferative potential, differentiation capacity, and angiogenic and immunomodulatory effects, as well as being a relevant and widely used cell type in this field [30,31,32].

## 2. Materials and Methods

### 2.1. Resins

The ideal resin for rapid prototyping of bioreactor parts that are in direct contact with cells is printable with a high resolution, transparent (optical observation), biocompatible (no cytotoxic effects), and heat resistant to enable steam sterilization. Since a resin that covered all of these requirements was not available, we utilized photopolymers that could represent one or several of these properties at an acceptable level (Table 1). All resins by formlabs (Somerville, MA, USA).

Black and Clear resins represent standard materials without special properties but should allow high-resolution printing. The chemical composition of both materials according to the manufacturer include 55–75% w/w urethane dimethacrylate, 15–25% w/w methacrylate monomers, and <0.9% w/w phenyl bis(2,4,6-trimethylbenzoyl)-phosphine oxide [37].

The dental resins (Dental SG and Dental LT) are both certified as biocompatible. Dental SG is based on ≥75% w/w ethoxylated bisphenol A dimethacrylate, 30–50% w/w 7,7,9(or 7,9,9)-trimethyl-4,13-dioxo-3,14-dioxa-5,12-diazahexadecane-1,16-diyl bismethacrylate, and <10% w/w phenyl bis(2,4,6-trimethylbenzoyl)-phosphine oxide [38]. Dental LT is composed of 50–75% w/w 7,7,9(or 7,9,9)-trimethyl-4,13-dioxo-3,14-dioxa-5,12-diazahexadecane-1,16-diyl bismethacrylate, 10–20% w/w 2-hydroxyethyl methacrylate, <10% w/w reaction mass of bis(1,2,2,6,6-pentamethyl-4-piperidyl) sebacate and methyl 1,2,2,6,6-pentamethyl-4-piperidyl sebacate, 1–5% w/w diphenyl(2,4,6-trimethylbenzoyl)phosphine oxide, 0.1–1% w/w acrylic acid, monoester with propane-1,2-diol, <10% w/w ethylene dimethacrylate, 0.1–1% w/w 2-hydroxyethyl acrylate, and < 0.1% w/w mequinol, 4-methoxyphenol, hydroquinone monomethyl ether [39].

The High Temp resin was developed to resist high temperatures up to 230 °C. According to the manufacturer, it was synthesized from 15–25% w/w (2,4,6-trioxo-1,3,5-triazine-1,3,5(2H,4H,6H)-triyl)tri-2,1-ethanediyl triacrylate, 40–60% w/w acrylate monomers, and 25–45% w/w urethane dimethacrylate [40].

Flexible was also reported to have a Vicat softening point of 230 °C being composed of 50–70% w/w urethane dimethacrylate, 30–40% w/w methacrylate monomers, and < 0.9% w/w diphenyl(2,4,6-trimethylbenzoyl) phosphine oxide [41].

### 2.2. Additive Manufacturing

The polymers were printed with the SLA desktop printer Form 2 (formlabs) with an XY-resolution of 25 µm, laser spot size of 140 µm, and layer thickness of 25–300 µm. Additionally, the High Temp resin was also coated with Parylene-F-VT4 (layer thickness 10 µm; Diener, Ebhausen, Germany).

To evaluate the printing performance regarding the reproducibility of complex features, level of detail, and surface structure, a test model file (formlabs) with patterns of increasing complexity was used. The dimensions of the test model were: 16.5 mm × 25.5 mm with details from 0.1 mm to 0.8 mm and slots from 0.4 mm to 1.2 mm, as well as 5.7 mm × 6.1 mm × 11.7 mm with a digit height of 2.3 mm. The CAD model for the cytotoxicity testing was generated with the Solid Works^®^ 2018 (Dassault Systems Solid Works Corp, Waltham, MA, USA) CAD package, transferred into STL files, and uploaded onto the 3D printer using PreForm software. The models were printed with a layer thickness of 100 µm since this is the best resolution of which all resins were capable (the thinnest possible layer thickness of the used resins: High Temp: 25 µm, Clear: 25 µm, Dental SG: 50 µm, Dental LT: 100 µm, Black: 25 µm, Flexible: 50 µm). To avoid potential cross-contamination, separate print platforms were used to ensure that no cytotoxic residuals were transferred.

### 2.3. Post-Processing

After printing, the printed parts were washed and cured according to the manufacturer’s instructions and observed with an optical microscope (VHX 5000 3D, Keyence Corporation, Osaka, Japan). The support structure was removed before washing. Washing was done in two baths with new isopropanol ≥98% each for the High Temp and Dental SG for 2 × 5 min and for each of the other resins for 2 × 10 min. After washing, the prints were allowed to air-dry. Afterwards, UV-curing was performed with a UVA-Cube 100 (Dr. Hönle AG, UV-Technologie, Gräfelfing/Munich, Germany) using a Dr. Hönle Strahler UV 150 F. The UV 150 F has a broadband spectrum from 250 nm to 600 nm with a relative intensity of 50% at 405 nm. The curing-time was based on formlabs’ recommendations [42].

### 2.4. Sterilization

High Temp, Dental LT, and Dental SG were sterilized by steam sterilization for 20 min at 121 °C in an autoclave (Varioklav 500E, Thermo Scientific, Waltham, MA, USA). Clear, Black, and Flexible did not withstand autoclavation and, therefore, were sterilized with UV-light (254 nm wavelength, 30 min each side).

### 2.5. Cell Culture

The use of human tissue was approved by the Ethics Committee of the Medical University Vienna, Austria (EK Nr. 957/2011, 30 January 2013), and all donors (female, 50–65 years old) gave written consent. Human adipose-derived MSCs were isolated within 8 h after surgery as described before [43]. MSCs were cultivated in standard medium composed of MEM alpha (Thermo Fisher Scientific, Waltham, MA, USA), 0.5% gentamycin (Lonza, Basel, Switzerland), 2.5% human platelet lysate (PL BioScience, Aachen, Germany), and 1 U/mL heparin (Ratiopharm, Ulm, Germany) in a humidified incubator at 37 °C, 5% CO_2_ and cryo-preserved in liquid nitrogen as described before (Neumann et al., 2014). Upon use, MSCs were thawed at passage 2 or 3 and subcultivated once. Then, MSCs were seeded at 2000 cells/cm^2^ in 24 well plates (TPP, Trasadingen, Switzerland) with 2 mL standard medium (n = 4). For the cytotoxicity testing, each resin was printed in the shape of discs (diameter of 5 mm, height of 2 mm). One disc per well was added and cells together with the discs were cultivated for 4 days. MSCs without additional discs served as the control.

### 2.6. Viability Assays

After 4 days of incubation with the printed photopolymers, the resazurin-based TOX8 kit (Sigma Aldrich/Merck KGaA, Darmstadt, Germany) and MTT viability assays were performed to assess the cytotoxicity of each resin. The TOX8 assay was used according to the manufacturer’s instructions. Fluorescence intensity at 560/590 nm was determined using a plate reader (Tecan, Männedorf, Switzerland) after 2 h incubation at 37 °C. For the MTT assay, the cells were covered with MTT solution (0.5 mg/mL in phosphate-buffered saline; both Sigma Aldrich). After 4 h of incubation at 37 °C, 10% sodium lauryl sulfate (Sigma Aldrich) solution was added and incubated overnight. Absorption was measured with the plate reader at 570 and 630 nm and differences between 570 and 630 nm calculated. The viability was normalized to values of the control.

### 2.7. Live/Dead Cell Staining

The viability of cells was visualized with calcein-AM (acetoxymethyl ester) and propidium iodide (PI) staining. Briefly, samples were stained with calcein-AM (4 μM) and PI (8 μM). After washing with PBS, samples were investigated with fluorescence microscopy (Leica DM IL LED with LeicaEL6000, both Leica Microsystems GmbH, Wetzlar, Germany).

### 2.8. Statistical Analysis

Statistical analysis was performed using GraphPad Prism Version 6.01 (GraphPad Software, La Jolla, CA, USA). Data are represented as the mean ± standard deviation (SD). Multiple comparisons against the control values were performed by ordinary one-way ANOVA followed by Dunnett’s multiple comparison test. Significance was accepted at *p* ≤ 0.05.

## 3. Results

### 3.1. Additive Manufacturing

**Figure 1:**
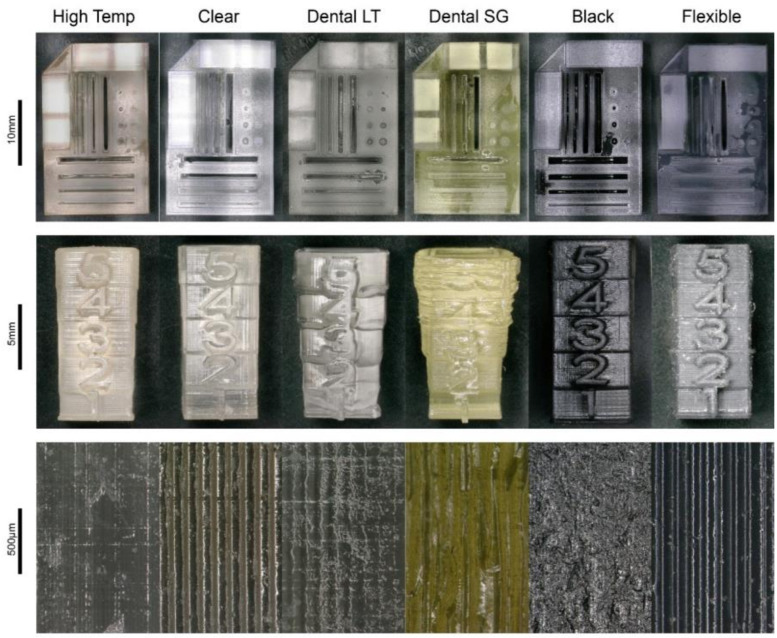
Results of the 3D prints of the photopolymers High Temp, Clear, Dental LT, Dental SG, Black, and Flexible. Top: test print for evaluation of the reproducibility of printing complex features. Centre: test print for evaluation of the level of detail. Bottom: optical enlargement of the surface structure of the different photopolymers.

Printing of the test model with complex structures and features revealed that Dental SG, Dental LT, and Flexible did not show sufficient reproducibility of features and levels of detail (Figure 1). In contrast, High Temp, Clear, and Black displayed a satisfactory degree of detail and accuracy. Regarding the surface finish, High Temp, Dental LT, and Black displayed relatively smooth surfaces, whereas Clear, Dental SG, and Flexible showed rough surfaces with grooves.

### 3.2. Cytotoxicity Testing

**Figure 2:**
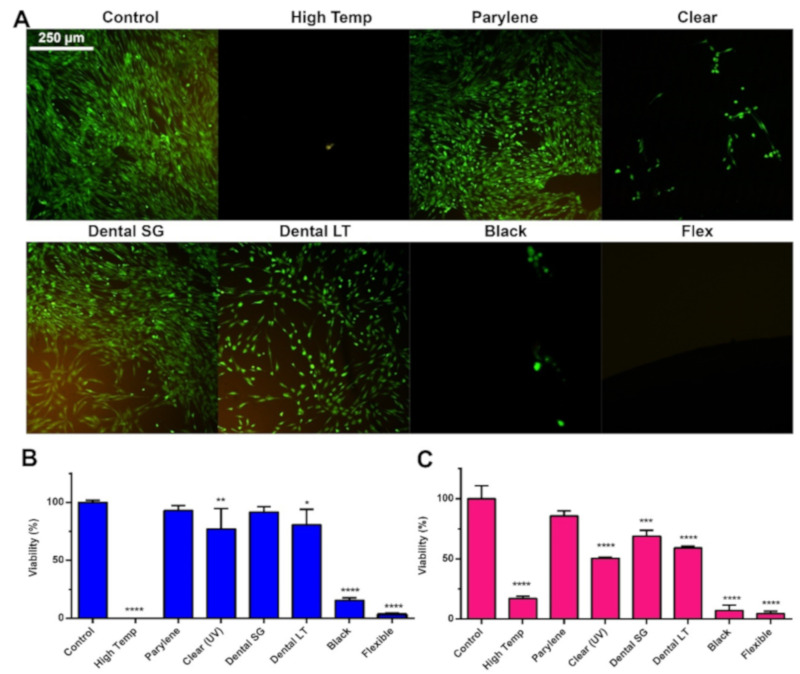
Cytotoxicity testing of resins for additive manufacturing. (**A**) Calcein-AM and PI staining, (**B**) TOX8 viability assay, and (**C**) MTT viability assay of MSCs after 4 days of cultivation with direct contact with the different resins. Data are given as mean ± SD (n = 4). * *p* < 0.05; ** *p* < 0.01; *** *p* < 0.001; **** *p* < 0.0001.

To assess the cytotoxicity of the resins, discs were printed from the resins, and they were incubated with MSCs for 4 days. Afterwards, calcein-AM and PI (live/dead) stain were used, and two viability assays (TOX8 and MTT) were applied to observe adverse effects on the cells (Figure 2). Live/dead staining of the cells showed that almost no cells survived when cultivated with High Temp, Clear (gamma), Black, and Flexible. A few living cells were observed in Clear (UV). However, those marked viable seemed morphologically distinct from the control. The roundish morphology of these cells resembled rather detaching or dying cells. Incubation with Dental LT decreased the amount of viable MSCs compared to the control, yet the cellular morphology was similar to the beforementioned cell morphology. However, after incubation with Parylene-coated High Temp resin and Dental SG, the number of viable cells was similar to that of the control. A similar trend was observed with the viability assays. MSCs incubated with High Temp, Black, and Flexible displayed a viability between 0 and 25%. Dental SG performed similar to the control in TOX8 (91%, not significantly lower), but displayed decreased viability in MTT (68%, significantly lower). Only the Parylene-coated resin did not cause a significantly lower viability compared to the control in both assays (TOX8 93%, MTT 85%). The viability values for Clear and Dental LT varied between the assays, while these resins significantly decreased the viability of MSCs.

Considering these results, only the Parylene-coated resin showed no adverse effects on the cells. As Dental SG caused only a minor, non-significant decrease in viability, it represented a non-coated resin for the manufacturing of parts that are in direct contact with cells.

## 4. Discussion

Rapid prototyping of specialized, tailor-made, or miniaturized cell cultivation platforms or cell culture devices is a valuable tool in the development of bioreactors or of devices for the expansion of cells in engineering of advanced (3D) tissue or disease models. However, studies on the cytocompatibility of the required polymers and especially photopolymers for 3D printing by SLA have received only minor attention so far. For this study we used a formlabs Form 2 printer as it is an affordable desktop printer and easy to use in a standard laboratory environment. Consequently, we tested several resins by formlabs that seemed to be reasonable candidates for rapid prototyping of construction parts that are in direct contact with cell culture medium.

Regarding the accuracy, only High Temp, Clear, and Black showed sufficient resolution and reproducibility for the printing of complex structures. To evaluate the cytotoxicity of the photopolymers, we incubated MSCs directly with printed discs of the different photopolymers. Beyond that, the incubation was sustained for 4 days in contrast to the standard exposure time of 24 h (according to ISO 10993-5 [33]), considering that in an actual cell culture application, the cells would be in prolonged contact with the material as well. High Temp, Clear, Black, and Flexible clearly caused cytotoxic effects, leading not only to a reduced proliferation in the case of Clear, but to death of almost all cells. The High Temp resin was also found to be toxic in a previous study performed with HeLa cells [23]. Incubation with Clear or “Clear-conditioned” media was found to reduce proliferation across several species and several human tumor cell lines (SH-SY5Y, HepG2, HeLa, L929) [17,23,24,25]. However, dependent on cell type and postprocessing, the toxicity was not as pronounced as in our study [25]. No data on the biocompatibility of Black and Flexible are available so far. Incubation with the dental resins Dental LT and Dental SG significantly reduced the viability of MSCs, although both resins are classified as biocompatible. These contradictions might originate from different incubation periods for cytotoxicity testing. Despite the significantly reduced viability after 4 days of incubation, we still found cells qualitatively and quantitatively viable compared to the other clearly cytotoxic materials. After a typical test period of 24 h, the Dental-incubated cells resembled the control and might be evaluated as biocompatible. The longer the incubation, the clearer the gradual long-term effect accumulated. Predicting therefrom, a further prolonged incubation might also lead to further reduced cellular viability, in particular for Dental LT. However, the herein determined toxic effects are minor compared to the cytotoxicity of the High Temp resin and might be acceptable for rapid prototyping when addressing short-term cell culture setups. A summary of the results is depicted in Table 2.

As all tested photopolymers reduced the viability, we tested if coating with Parylene could shield MSCs from cytotoxic leachates. As High Temp is an autoclavable material with excellent optical resolution, we decided to coat High Temp with Parylene. In fact, coating with Parylene completely protected MSCs from the cytotoxic effects of the High Temp resin. In contrast to coating with wax, which has been reported to mitigate the toxicity of printed parts [44] (but only for about 40 h), Parylene was found to remove toxicity for at least 4 days in our study. Unfortunately, coating with Parylene is costly and not easily achievable in standard laboratories. Furthermore, the coating was found to detach after repeated autoclavation (after approximately 5–6 repetitions).

Considering the before mentioned drawbacks, coating with Parylene represents a viable option to shield cells from the cytotoxic effects of materials even for long-term in vivo application [45,46]. Besides coating with a biocompatible material, other options to mitigate toxicity of polymers for rapid prototyping are customizing the resin formulas and optimizing the polymerization and post-processing procedures [16]. However, customizing the formula of commercially available resins might be labor-intensive and not feasible for non-specialists. Optimization of the polymerization process can also increase the biocompatibility of a resin. For this, the polymerizing light source needs to be matched to the absorption of the photoinitiator [47]. However, this again requires customizing the initial resin. Other options to reduce the cytotoxicity of a resin is post-processing with supercritical carbon dioxide [48], sonication of the material in isopropanol [49], or 10 days of incubation in cell growth medium [24] to leach harmful chemicals.

## 5. Conclusions

None of the tested photopolymers can be considered as non-toxic. Although the dental resins are classified as biocompatible, we found adverse effects. Coating of the printed parts with Parylene completely protected MSCs from toxic effects in our study. However, if coating with Parylene is not an option, the dental resins might be acceptable for short-term testing of devices that are in direct contact with the cultivation medium, depending on the required resolution or absence of cytotoxic effects.

## Figures and Tables

**Table 1:** Properties of the photopolymers tested for rapid prototyping.

Photopolymer	Order Number	Properties
High Temp	FLHTAM02	Heat resistant up to 238 °C.
Clear	FLGPCL04	Optical transparency, high resolution
Dental SG	FLSGAM01	Class I Medical Device, biocompatible (not cytotoxic, no irritation, no sensitization) according to EN ISO 10993-5:2009 [33], ISO 10993-10:2010/(R)2014 [34])
Dental LT	FLDLCL01	Biocompatible according to EN-ISO 10993-1:2009/AC:2010 [35]
Black	FLGPBK04	High resolution
Flexible	FLFLGR02	High heat resistance, Vicat softening point of 230 °C

Data on the material properties were derived from the manufacturer’s material data sheets; the full list of material-parameters is available at the formlabs website [36], though the exact formulation is not publicly available.

**Table 2:** Summary of material and cytotoxicity testing of photopolymers.

Photopolymer	Translucent	Autoclavable	Reproducibility	Level of Detail	Cytotoxicity
High Temp	Medium	Yes	High	High	High
Clear	Yes	No	High	High	Medium
Dental SG	No	Yes	Low	Low	Very low
Dental LT	Medium	Yes	Medium	Medium	Low
Black	No	No	High	High	High
Flexible	No	No	Low	Low	High
